# Reduced Diet-induced Thermogenesis in Apolipoprotein A-IV Deficient Mice

**DOI:** 10.3390/ijms20133176

**Published:** 2019-06-28

**Authors:** Sydney Pence, Qi Zhu, Erin Binne, Min Liu, Haifei Shi, Chunmin C. Lo

**Affiliations:** 1 Department of Biomedical Sciences, Heritage College of Osteopathic Medicine, Diabetes Institute and Honor Tutorial College, Ohio University, Athens, OH 45701, USA; 2 Department of Biology, Miami University, Oxford, OH 45056, USA; 3 Department of Pathology and Laboratory Medicine, Metabolic Diseases Institute, University of Cincinnati, Cincinnati, OH 45215, USA

**Keywords:** brown adipose tissue, white adipose tissue, energy homeostasis, food intake, high-fat diet, dietary lipids, uncoupling protein 1, tyrosine hydroxylase, cold exposure

## Abstract

In the presence of dietary lipids, both apolipoprotein A-IV (ApoA-IV) production and brown adipose tissue (BAT) thermogenesis are increased. The effect of dietary lipid-induced AproA-IV on BAT thermogenesis and energy expenditure remains unknown. In the present study, we hypothesized that ApoA-IV knockout (ApoA-IV-KO) mice exhibited decreased BAT thermogenesis to affect energy homeostasis. To test this hypothesis, BAT thermogenesis in wildtype (WT) and ApoA-IV-KO mice fed either a standard low-fat chow diet or a high-fat diet (HFD) was investigated. When fed a chow diet, energy expenditure and food intake were comparable between WT and ApoA-IV-KO mice. After 1 week of HFD consumption, ApoA-IV-KO mice had comparable energy intake but produced lower energy expenditure relative to their WT controls in the dark phase. After an acute feeding of dietary lipids or 1-week HFD feeding, ApoA-IV-KO mice produced lower levels of uncoupling protein 1 (UCP1) and exhibited reduced expression of thermogenic genes in the BAT compared with WT controls. In response to cold exposure, however, ApoA-IV-KO mice had comparable energy expenditure and BAT temperature relative to WT mice. Thus, ApoA-IV-KO mice exhibited reduced diet-induced BAT thermogenesis and energy expenditure.

## 1. Introduction

Obesity has become a global epidemic, affecting more than 90 million people or almost 40% of adults in the U.S. [1]. In parallel, the incidence of type 2 diabetes mellitus, cardiovascular diseases, and certain types of cancer has also increased [2,3,4,5]. Consumption of a high-fat diet (HFD) promotes excess energy intake, leading to excess energy stored as fat and subsequent development of obesity [6]. Enhancement of energy expenditure and brown adipose tissue (BAT) thermogenesis increases triglyceride clearance, reduces hypertriglyceridemia and hypercholesterolemia, and protects against atherosclerosis development [7,8,9]. Increased BAT thermogenesis also improves insulin sensitivity and regulates glucose homeostasis [5,10,11]. Thus, stimulation of energy expenditure and BAT thermogenesis could have far-reaching health benefits in combatting obesity and obesity-related complications including diabetes and cardiovascular diseases [5,9,12,13].

Apolipoprotein A-IV (ApoA-IV) is one of the most abundant proteins (as much as 3% of the proteins) made by enterocytes and the brain in the presence of dietary lipids [14,15,16,17]. ApoA-IV presented on the surface of chylomicrons is secreted into the lymphatic system during lipid infusion. Subsequently, ApoA-IV is transferred to high-density lipoprotein or becomes a lipoprotein-free ApoA-IV during subsequent metabolism of chylomicrons [14,16]. The small intestine contributes approximately 60% of the plasma ApoA-IV pool [17]. Thus, dietary lipids are important stimulants of ApoA-IV production in the small intestine. ApoA-IV is a well-known protein with short-term satiating effects, along with its well-established roles in modulating cholesterol efflux, anti-atherosclerosis, lipid transport, glucose homeostasis, and anti-inflammation [16,18,19,20]. Because circulating ApoA-IV fails to cross the blood-brain barrier [21], it acts on vagal afferents to relay neuronal activation to the hindbrain to suppress food intake [22,23]. Central administration of ApoA-IV acts on hypothalamic nuclei to suppress food intake via the central melanocortin system [21,24,25]. Thus, peripheral and central ApoA-IV suppresses energy intake to control energy homeostasis.

Short-term consumption of dietary lipids elevates BAT thermogenesis through the activation of gut-brain-BAT neurocircuits in animals including humans to counteract the energy surplus [26,27,28]. The action of dietary lipids on sympathetic activity of BAT leads to release of neural norepinephrine, thereby stimulating BAT β3-adrenergic receptor signaling to increase lipolysis and activate uncoupling protein 1 (UCP1)-dependent BAT thermogenesis [5,29,30]. ApoA-IV knockout (ApoA-IV-KO) mice maintained on a low-fat diet have similar energy expenditure as wildtype (WT) control mice; whereas ApoA-IV-KO mice have reduced energy expenditure after a chronic consumption of HFD for 20 weeks [31]. The observations indicate that endogenous ApoA-IV may elevate energy expenditure. Previous reports have demonstrated that a short-term feeding of HFD for 1 week increases ApoA-IV secretion in the small intestine [32,33]. In contrast, dietary lipids fail to stimulate ApoA-IV production in the small intestine when rodents fed a HFD for a period longer than 2 weeks [32,34]. These findings suggest that chronic consumption of a HFD abolishes increased ApoA-IV production in WT mice. The involvement of endogenous ApoA-IV in the regulation of energy expenditure remains unknown. Thus, the objectives of the present experiments were sought to investigate if endogenous ApoA-IV induced by either acute feeding of dietary lipids or a short-term feeding of HFD enhanced BAT thermogenesis and energy expenditure. The hypothesis of this study was that ApoA-IV-KO mice exhibited reduced BAT thermogenesis to regulate energy homeostasis. In the present studies, feeding behavior, energy expenditure, expression of BAT thermogenic genes, and BAT thermogenesis in WT and ApoA-IV-KO mice in response to an acute feeding of dietary lipids and a short-term feeding of HFD for 1 week were investigated. Additionally, potential effect of ApoA-IV on cold- induced BAT thermogenesis was explored using WT and ApoA-IV-KO mice.

## 2. Results

### 2.1. Experiment 1. Energy Expenditure, Respiratory Quotient (RQ), and Food Intake of Chow-fed Mice

**Figure 1:**
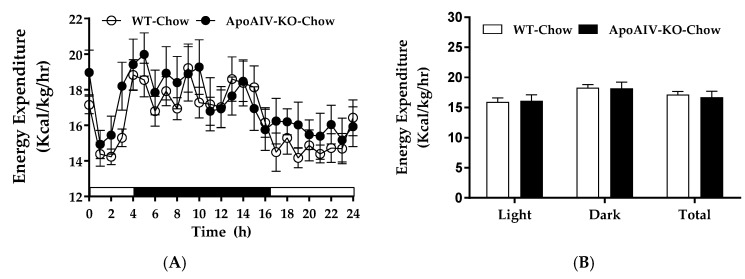

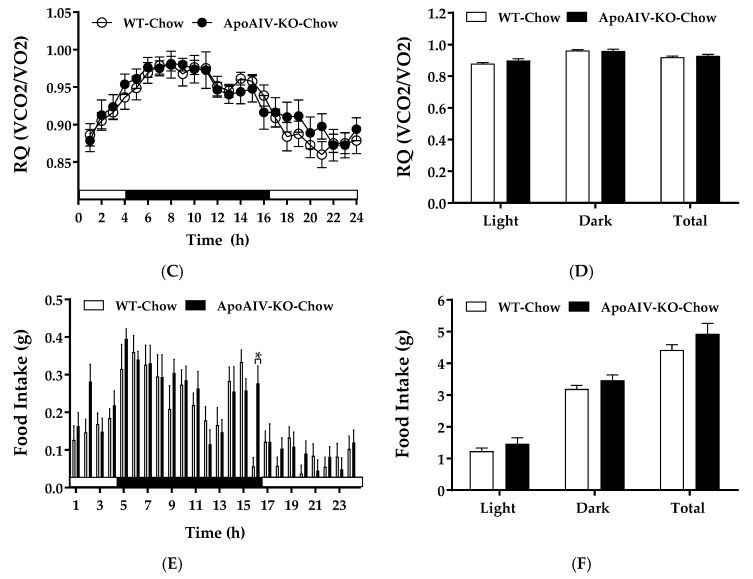
Energy expenditure and intake in WT and ApoA-IV-KO mice fed a chow diet. Hourly energy expenditure (**A**), average energy expenditure in the light and dark phases (**B**), hourly respiratory quotient (**C**), average respiratory quotient in the light and dark phases (**D**), meal size (**E**), and food intake (**F**) of chow-fed WT and ApoA-IV-KO mice. Data are expressed as mean ± SEM. *n* = 8 per group. * Represent significant differences relative to WT mice (*p* < 0.05).

When fed a standard chow diet, WT and ApoA-IV-KO mice had comparable energy expenditure (Figure 1A,B) and RQ (Figure 1C,D) during both light and dark phases. Although ApoA-IV-KO mice had a significant increase in meal size compared with WT mice at one timepoint towards the end of the dark phase (Figure 1E), the overall food intake (Figure 1F) and body weights (Table 1) were similar between these two groups. 

Fat mass of interscapular BAT, epididymal white adipose tissue (EWAT), and inguinal white adipose tissue (IWAT) were comparable between two groups (Table 1). ApoA-IV-KO mice tended to have increased fasting glucose compared to WT mice (*p* = 0.1436). Plasma lipid and hormone levels were comparable between WT and ApoA-IV-KO mice when they were maintained on the chow diet (Table 1).

### 2.2. Experiment 2. Expression of BAT Thermogenic Protein of Mice with Acute Feeding of Dietary Lipids

**Figure 2:**
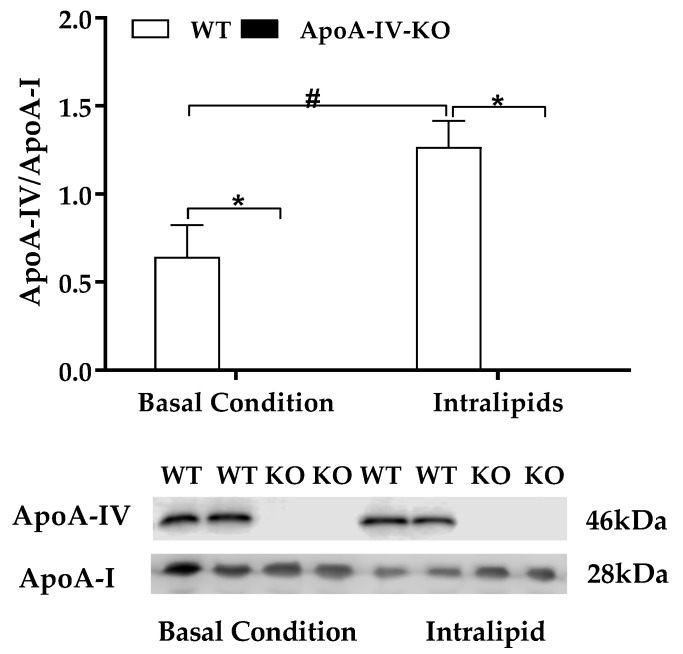
Plasma levels of apolipoproteins. Plasma ApoA-IV/ApoA-I protein ratio in 5-h fasted mice and in mice treated with Intralipid. Data are expressed as mean ± SEM for 6 animals per group. * Represent significant difference relative to WT mice under the same feeding condition (*p* < 0.05). # Represent significant difference relative to basal condition within the same genotype (*p* < 0.05).

Dietary lipid intake increases ApoA-IV produced by small intestine [14,15,16,17]. After either 5-h fast or an intralipid gavage, plasma ApoA-IV protein was detected in WT mice, whereas plasma ApoA-IV was absent in ApoA-IV-KO mice (Figure 2). When mice were maintained on a chow diet, ApoA-IV-KO mice produced similar basal level of plasma ApoA-I protein relative to WT mice after 5-h fasting (Figure 2). After an intralipid gavage, ApoA-IV-KO mice produced a greater level of ApoA-I protein than WT mice (*p* < 0.05; Figure 2), maybe to compensate the lack of ApoA-IV. Dietary lipids induced a significantly greater ApoA-IV/ApoA-I ratio than 5-h fast in WT mice (*p* < 0.05, Figure 2).

**Figure 3:**
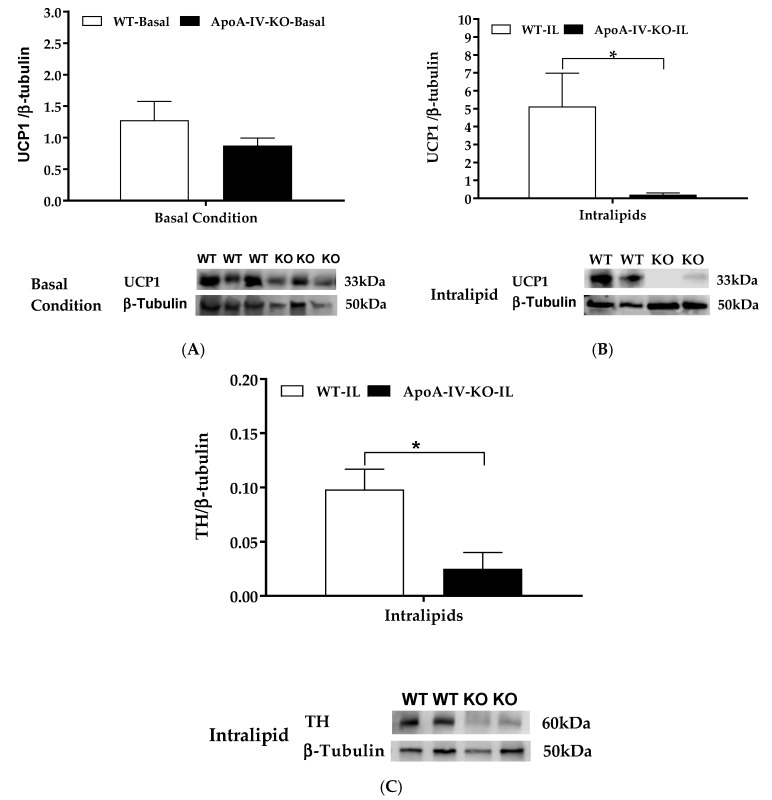
Thermogenic protein in the BAT of WT and ApoA-IV-KO mice in 5-h fasted mice or after intra-gastric gavage of dietary lipids. BAT UCP1/β-tubulin protein ratio in 5-h fasted mice (**A**) and in mice treated with 100 µL Intralipid (**B**). Tyrosine hydroxylase (TH) β-tubulin protein ratio in the BAT of WT and ApoA-IV-KO mice treated with 100 µL Intralipid (**C**). Data are expressed as mean ± SEM. *n* = 8 per group for the basal condition, and *n* = 6 per group for the lipid-induced group. * Represent significant differences relative to WT mice under the same feeding condition (*p* < 0.05).

Dietary lipid intake activates gut-brain-BAT neurocircuits to induce BAT thermogenesis [26,27,28]. UCP1 in the mitochondrion of BAT assists heat production and is used as a marker for BAT thermogenesis [5]. Tyrosine hydroxylase is the rate-limiting enzyme for the synthesis of nor-epinephrine in peripheral sympathetic neurons that is released when sympathetic activity is stimulated [35]. Thus, the presence of tyrosine hydroxylase protein can be used to indicate neural norepinephrine release and as a marker for sympathetic innervation [36]. To determine UCP1 protein expression at the basal level, 100 µg extracted BAT protein per lane were loaded onto a gel. When they were maintained on a chow diet, BAT UCP1 protein expression in ApoA-IV-KO mice tended to be lower than that in WT mice, but the difference was not significant (Figure 3A). In the present experiment, the basal level of tyrosine hydroxylase protein in the BAT was too low to be detected, even though 100 µg extracted BAT protein per lane was loaded onto a gel (data not shown). Intralipid is a fat emulsion, providing a ready source of triglycerides for uptake by the BAT during thermogenesis [37]. To investigate whether lipid-induced ApoA-IV elevates diet-induced BAT thermogenesis through induction of norepinephrine synthesis, BAT thermogenic UCP1 and tyrosine hydroxylase proteins in WT and ApoA-IV-KO mice were determined. Dietary lipids stimulated BAT UCP1 expression in WT mice. After an intragastric gavage of dietary lipids, ApoA-IV-KO mice had significantly lower UCP1 and tyrosine hydroxylase proteins than WT mice (*p* < 0.05, Figure 3B,C). These observations suggest that the ApoA-IV-KO mice produced lower lipid-induced BAT thermogenic protein UCP1 through induction of norepinephrine synthesis than WT mice.

### 2.3. Experiment 3.1. Energy Expenditure, RQ, and Food Intake of Mice Fed a HFD for 1 Week

**Figure 4:**
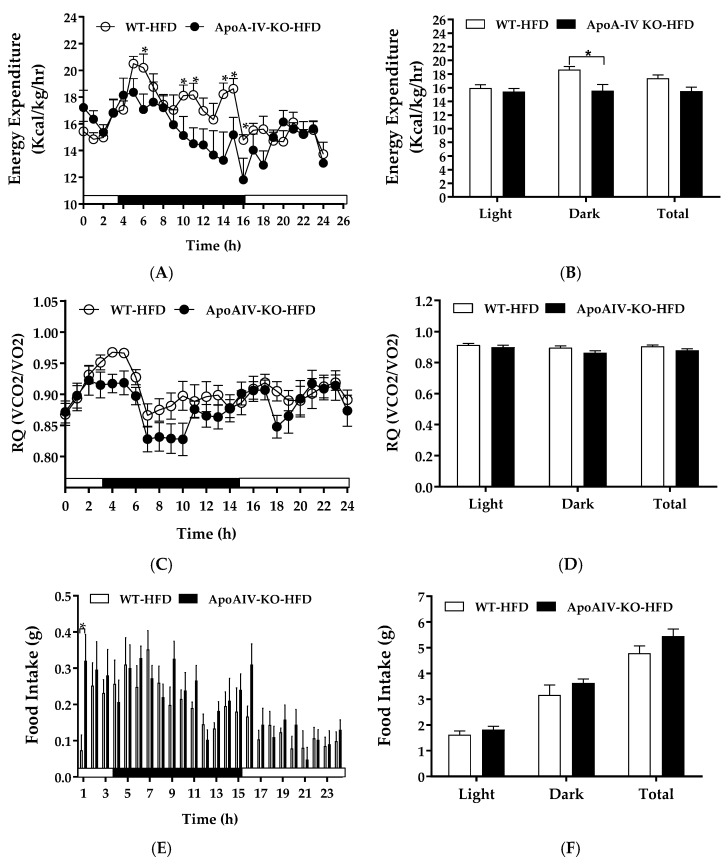
Energy expenditure and intake in WT and ApoA-IV-KO mice fed a high-fat diet (HFD) for 1 week. Hourly energy expenditure (**A**), average energy expenditure in the light and dark phases (**B**), hourly respiratory quotient (RQ, **C**), average RQ in the light and dark phases (**D**), meal size (**E**), and food intake (**F**) of WT and ApoA-IV-KO mice. Data are expressed as mean ± SEM. *n* = 8 per group. * Represent significant differences relative to the WT mice (*p* < 0.05).

Diet-induced BAT thermogenesis contributes to whole-animal energy expenditure [5]. To examine if endogenous ApoA-IV regulates energy homeostasis in response to 1-week HFD feeding, energy expenditure and food intake of WT and ApoA-IV-KO mice were determined after 1-week HFD feeding. Relative to the WT mice, ApoA-IV-KO mice fed a HFD exhibited lower energy expenditure at some timepoints during the dark phase as well as lower average energy expenditure in the dark phase (*p* < 0.05, Figure 4A,B). Additionally, RQ of these ApoA-IV-KO mice tended to be lower than WT mice, but the difference was not statistically different (Figure 4C,D). The findings indicate that these ApoA-IV-KO mice might utilize more fatty acids as energy substrates. 

ApoA-IV-KO mice exhibited increased hourly food intake with respect to their WT control group at one timepoint and their total food intake was comparable to WT mice (Figure 4E,F). No significant difference in body weight or fat mass was found between ApoA-IV-KO and WT mice (Table 2). Thus, HFD reduces energy expenditure in ApoA-IV-KO mice independent of food intake.

### 2.4. Experiment 3.2. Expression of BAT Thermogenic Protein and Genes of Mice Fed a HFD for 1 Week

**Figure 5:**
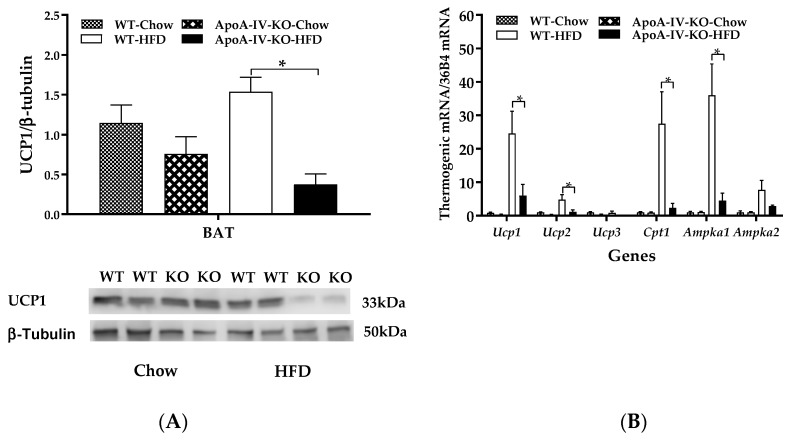
Thermogenic protein and gene expression in BAT of chow-fed and HFD-fed mice. Expressions of BAT UCP1 β-tubulin protein ratio (**A**) and BAT thermogenic mRNA/36B4mRNA (**B**) in 5-h fasted WT and ApoA-IV-KO mice after a 1-week feeding of either a standard chow diet or a HFD. Data are expressed as mean ± SEM for 6 animals per group. Values with asterisks represent significant differences relative to the WT mice (*p* < 0.05).

To understand whether impaired BAT thermogenesis results in reduced energy expenditure in HFD-fed Apo-AIV-KO mice, expressions of BAT thermogenic proteins and genes of WT and ApoA-IV-KO mice were measured using immunoblots and qPCR, respectively. When mice were maintained on a chow diet, BAT UCP1 protein expression of ApoA-IV-KO mice was comparable to their WT control mice (Figure 5A). In contrast, after 1-week HFD, UCP1 proteins in the BAT of ApoA-IV-KO mice were significantly lower than that in WT mice (*p* < 0.05, Figure 5A). Because phospho-AMP-activated protein kinase (AMPK) protein was not detected in the BAT of HFD-fed mice, thermogenic gene expressions in the BAT were determined in the present study. ApoA-IV-KO mice exhibited lower *Ucp1*, *Ucp2*, *Cpt1*, and *Ampka1* expressions than WT mice (*p* < 0.05, Figure 5B). In addition, expressions of *Ucp3* and *Ampka2* in the BAT of ApoA-IV-KO mice tended to be lower than WT mice, but the differences in these expressions were not statistically significant. These findings indicated that ApoA-IV-KO mice had impaired UCP1-dependent BAT thermogenesis after a short-term feeding of HFD.

### 2.5. Experiment 3.3. Plasma Parameters and Adipocyte Histology of Mice Fed a 1-week HFD

**Figure 6:**
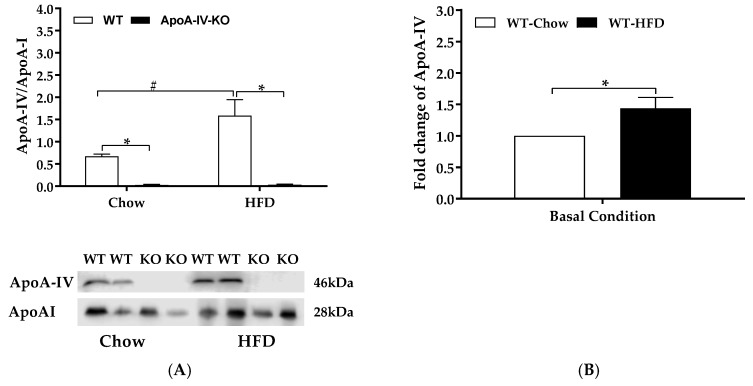
Plasma levels of apolipoproteins. Plasma ApoA-IV and ApoA-I of WT and ApoA-IV-KO mice after 1-week feeding of either a standard chow diet or a high-fat diet (HFD). Plasma ApoA-IV/ApoA-IV protein ratio (**A**) and the fold change of ApoA-IV in HFD-fed relative to chow-fed WT mice (**B**). Plasma of WT and ApoA-IV-KO mice was collected after 5-h fast. Data are expressed as mean ± SEM for 6 animals per group. * Represent significant differences relative to the WT mice under the same feeding condition (*p* < 0.05). # Represent significant differences relative to basal condition within the same genotype (*p* < 0.05).

WT mice and ApoA-IV-KO mice produced similar plasma ApoA-I protein when fed the chow diet and after 1-week feeding of HFD (Figure 6A). Additionally, compared with the chow-fed WT mice, HFD-fed WT mice produced 1.5 folds of plasma ApoA-IV (Figure 6B). Consequently, HFD-fed WT mice produced a greater ApoA-IV/ApoA-I protein ratio than chow-fed WT mice (*p* < 0.05, Figure 6A). Plasma ApoA-IV level in ApoA-IV-KO mice was not detected (Figure 6A) and confirmed that the ApoA-IV KO mice failed to secrete ApoA-IV into the circulation.

**Figure 7:**
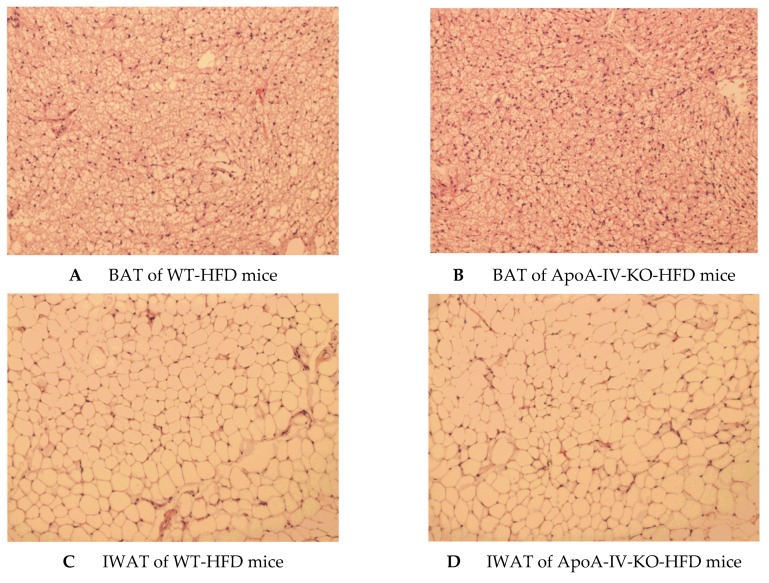
Histology of brown adipose tissue (BAT) of WT mice (**A**) and ApoA-IV-KO mice (**B**), and inguinal white adipose tissue (IWAT) of WT mice (**C**) and ApoA-IV-KO mice (**D**) determined by H&E staining. WT and ApoA-IV-KO mice (*n* = 4 per group) received a high-fat diet for 1 week, and BAT and IWAT of 5-h fasted mice were collected in 10% formalin solution and processed for histology.

After 1-week HFD feeding, plasma levels of triglyceride, glucose and leptin of ApoA-IV-KO mice were comparable to those parameters of WT mice (Table 2). In contrast, plasma levels of cholesterol and insulin of ApoA-IV-KO mice were significantly lower than those of WT mice (*p* < 0.05, Table 2). In addition, after a 1-week HFD, BAT and IWAT of WT and ApoA-IV-KO mice had similar weights, contained similar numbers and sizes of adipocytes (Figure 7 and Table 3).

### 2.6. Experiment 4. Energy Expenditure and BAT Temperature of Chow-fed Mice during Cold Exposure

**Figure 8:**
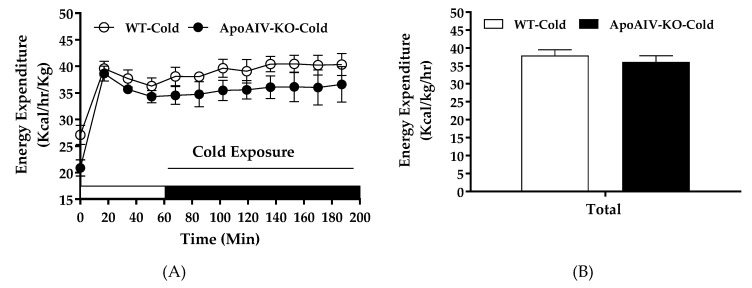

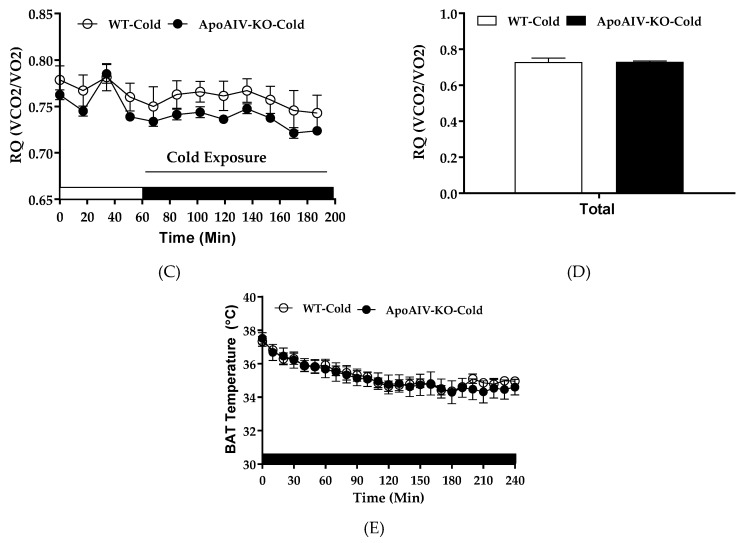
Energy expenditure, respiratory quotient (RQ), and BAT temperature in 5-h fasted WT and ApoA-IV-KO mice exposed to cold temperature. Hourly energy expenditure (**A**), average energy expenditure (**B**), hourly RQ (**C**), average RQ (**D**) of ApoA-IV-KO and WT mice (*n* = 8 per group) in cold-exposed environment. BAT temperature (**E**) of food-deprived and cold-exposed WT and ApoA-IV-KO mice (*n* = 6 per group) measured with an implanted temperature probe. Data are expressed as mean ± SEM.

Cold exposure activates sympathetic neural input to increase UCP1-dependent thermogenesis and elevates whole-body energy expenditure [5]. To investigate if dampened sympathetic activity in BAT of ApoA-IV-KO mice, indicated by low expression of tyrosine hydroxylase (Figure 3C), results in reduced BAT thermogenesis and energy expenditure, their energy expenditure and BAT temperature were monitored during acute cold exposure. After 5-h fast, ApoA-IV-KO mice exhibited comparable body temperature (38.0 ± 0.12 °C) relative to WT mice (38.2 ± 0.19 °C). In cold-exposed environment, hourly energy expenditure, RQ, and BAT temperature of ApoA-IV-KO mice were comparable to those parameters of WT mice (Figure 8A,C). In addition, the average of hourly energy expenditure and RQ of ApoA-IV-KO mice across the entire cold exposure period were comparable to those parameters of WT mice (Figure 8B,D). Furthermore, ApoA-IV-KO mice also had similar BAT temperature relative to WT mice in cold-exposed environment (Figure 8E). Therefore, chow-fed WT and ApoA-IV-KO mice had comparable cold-exposed energy expenditure and RQ with similar BAT temperature.

## 3. Discussion

Although endogenous ApoA-IV stimulated by dietary lipid consumption is a well-known satiety signal, the effect of ApoA-IV on the regulation of BAT thermogenesis remains elusive. These experiments test the hypothesis that ApoA-IV-KO mice exhibited reduced BAT thermogenesis to regulate energy homeostasis. These experiments demonstrated that chow-fed ApoA-IV-KO mice had comparable basal level of UCP1-dependent BAT thermogenesis relative to their WT control groups. Consistent with previous reports [31,38], the present study showed that chow-fed ApoA-IV-KO mice had normal energy expenditure and RQ, and did not alter total food intake when WT and ApoA-IV-KO mice were maintained on a chow diet. The possible explanation for the nonsignificant difference in energy expenditure between two genotypes may be due to low production of endogenous ApoA-IV in the WT mice. After 5-h fast, chow-fed WT and ApoA-IV-KO mice exhibited comparable BAT temperature and body temperature (Experiment 4), and comparable levels of energy expenditure, body weight, fat mass, and plasma parameters (Experiment 1). These findings suggest that ApoA-IV-KO mice have normal energy homeostasis when they are maintained on a standard chow diet.

Mice have a minimal BAT activity at thermoneutrality (28–30 °C), which is the range of ambient temperatures without regulatory changes in metabolic heat production or heat loss [5]. One study has compared thermoregulatory curves of single housed mice, and concluded 26–28 °C as the optimal housing temperature to achieve thermoneutrality for mice with body weights between 25 and 40 g [39]. In the present study, when the housing temperature for animals at 25 °C was slightly lower than their thermoneutrality, the BAT activity of animals might be activated to produce extra heat to defend their body temperature. Since there was no significant difference in body temperature or BAT temperature between the two genotypes, the whole-body energy expenditure in ApoA-IV-KO mice was comparable to their WT control group although BAT thermogenesis was supposed to be elevated at 25 °C. Acute cold exposure induces a maximal effect of sympathetic activity in BAT and increases whole-body energy expenditure in animals via non-shivering thermogenesis [5]. The current experiments showed that 5-h fasted ApoA-IV-KO mice had comparable BAT temperature and whole-body energy expenditure relative to their WT control group during acute cold exposure with food deprivation, suggesting that ApoA-IV-KO mice well maintain BAT temperature and energy expenditure in acute cold-evoked environment.

Following 20 weeks of HFD, ApoA-IV-KO mice produce lower energy expenditure in the dark phase independent of food intake and locomotor activity [31]. The present study demonstrated that HFD-fed ApoA-IV-KO mice had a significant reduction in hourly energy expenditure in the dark phase. The findings suggested that when animals consumed more HFD in the dark phase, the induction of ApoA-IV enhanced BAT thermogenesis in WT mice, and consequently elevated whole-body energy expenditure. Feeding a HFD tended to increase ApoA-IV level in WT mice, resulting in elevated UCP1-dependent thermogenesis. Since the difference in BAT and body temperatures between chow- and HFD-fed ApoA-IV-KO mice remains unclear, the effects of ApoA-IV on the regulation of BAT and body temperatures are needed to be investigated. In the current experiment, ApoA-IV-KO mice tended to use more fatty acids as energy substrate than WT mice, indicated by lower RQ (Figure 4C,D), suggesting that ApoA-IV-KO mice might have increased intracellular lipolysis to enhance fatty acid oxidation. Further investigation of ApoA-IV in the regulation of intracellular lipid metabolism in BAT is required. Consistent with the findings of ApoA-IV as a short-term satiating protein [22,23], the current experiment showed that HFD-fed ApoA-IV-KO mice exhibited comparable food intake relative to HFD-fed WT mice, contributing to similar body weight, fat mass, and the sizes and numbers of adipocytes at BAT and IWAT between HFD-fed WT and ApoA-IV-KO mice. Energy expenditure is controlled by basal metabolic rate, physical activity, thermoregulation, and the thermic effect of food [40]. Thus, HFD-fed ApoA-IV-KO mice had the impaired energy expenditure in the dark phase independent of daily caloric intake.

ApoA-IV is expressed in the small intestine and the liver in rodents [40]. In response to dietary lipids, the small intestine contributes to the major proportion of increased circulating ApoA-IV, whereas hepatic production of ApoA-IV is not altered [32,41]. The present study showed that acute gavage of dietary lipids and short-term HFD feeding increased plasma ApoA-IV in WT mice. In response to excess caloric intake, diet-induced thermogenesis occurs in BAT [5,42]. Lipid-induced norepinephrine from sympathetic nerves in BAT leads to stimulation of UCP1-dependent BAT thermogenesis [5,36]. In the present study, acute feeding of dietary lipids lowered productions of UCP1 and tyrosine hydroxylase in ApoA-IV-KO mice than those in their WT controls, suggesting that the absence of lipid-induced ApoA-IV may attenuate UCP1-dependent thermogenesis through the reduction of norepinephrine synthesis. The current experiments demonstrated that feeding a HFD tended to stimulate greater BAT thermogenesis than a standard chow diet in WT mice, but the difference in BAT thermogenesis between chow diet- and HFD-fed mice was not significant. It is possible that after 5-h fast, HFD-fed WT mice produce low level of ApoA-IV and consequently their BAT thermogenesis is comparable to chow-fed WT mice. In addition, ApoA-IV-KO mice exhibited lower BAT thermogenesis than WT mice after a short-term feeding of HFD. UCP proteins including UCP1, UCP2, and UCP3 are expressed in BAT [43,44,45,46]. Carnitine palmitoyltransferase 1 (CPT1) is a key enzyme of fatty acid oxidation which facilitates the transfer of fatty acids into the mitochondrion for oxidation [47]. Activation of AMPK pathway increases CPT1 activity and UCP1-induced BAT thermogenesis [48,49,50]. Because phospho AMPK proteins in the BAT was too low to be detected using immunoblots, the present experiments showed that ApoA-IV-KO mice had reduced expressions of genes coding AMPK, CPT1 and UCP1. The observations suggest that HFD-fed ApoA-IV-KO mice exhibited reduced actions of AMPK, CPT1 and UCP1 pathways. HFD increases gene expression of *Ucp1*, but not *Ucp2* or *Ucp3*, in the BAT of WT mice [43,51]. ApoA-IV-KO mice had reduced BAT *Ucp2* expression and non-detectable *Ucp3* expression, suggesting that the absence of ApoA-IV itself might be involved in the regulation of *Ucp2* and *Ucp3* expression in the BAT independent of HFD. The current experiments showed that HFD-fed ApoA-IV-KO mice had a reduction in expression of the BAT thermogenic genes compared with their WT control group. Presumably, they also had reduced BAT thermogenesis during HFD feeding.

The present experiment showed that ApoA-IV-KO mice had comparable levels of plasma triglyceride when animals were maintained on either a chow diet or 1-week HFD. ApoA-IV-KO mice have normal fat absorption, very-low density lipoprotein (VLDL) production and fatty acid uptake by BAT, except for delayed chylomicron clearance [31,39,52]. Thus, ApoA-IV-KO mice exhibited normal transport of TG. Plasma cholesterol and insulin of ApoA-IV-KO mice tended to be lower than WT mice when they were maintained on a chow diet, but the difference between two genotypes was not significant. Consistent with a previous observation [39], HFD-fed ApoA-IV-KO mice had reduced levels of plasma cholesterol compared with their WT control group in the present study. The possibility for the reduced cholesterol in ApoA-IV-KO mice might be due to the deficiency of ApoA-IV involved in the regulation of hepatic cholesterol transport and/or cholesterol efflux [18,53]. These effects of ApoA-IV might influence lipid accumulation within adipocytes. ApoA-IV alters plasma glucose and insulin in a hyperglycemic condition, and ApoA-IV-KO mice have impaired insulin production in response to a glucose challenge [54]. Consistent with a previous report [53], the present experiment showed that, compared to WT mice, ApoA-IV-KO mice tended to have increased fasting glucose but comparable basal levels of insulin and cholesterol when animals are maintained on a chow diet with 59% carbohydrates and 6% fat (Table 1). After a short-term feeding of a HFD with 45% carbohydrates and 20% fat, however, ApoA-IV-KO mice had comparable level of fasting glucose and reduced level of insulin and cholesterol compared to WT mice (Table 2), possibly due to lack of ApoA-IV-induced insulin. Insulin enhances fatty acid uptake by adipose tissues [55]. The lower insulin levels in HFD-fed ApoA-IV-KO mice would result in reduced lipid storage and smaller mass of white adipose tissues relative to WT mice if they receive a HFD for a longer period. In the currently study, the number and size of brown and white adipocytes were not statistically different between ApoA-IV-KO and WT mice after 1-week HFD feeding. The findings suggest that reduced levels of insulin and cholesterol might indirectly influence HFD-induced BAT thermogenesis in ApoA-IV-KO mice. The findings also suggest that endogenous ApoA-IV is involved in the regulation of glucose and lipid metabolism in response to feeding a chow diet with high content of carbohydrates or a diet high in lipids. Further study of the cholesterol transport and lipid uptake in ApoA-IV-KO and WT mice is warranted.

Activating BAT in humans has a great potential to combat obesity, insulin resistance, and cardiovascular diseases [9,56,57]. In view of the observations using the global deficiency of ApoA-IV mice, lipid-induced endogenous ApoA-IV may increase UCP1-dependent BAT thermogenesis. Because dietary lipids increase levels of ApoA-IV in the small intestine and in the hypothalamus [14,15,58], further investigations should be conducted to determine whether peripheral ApoA-IV and/or hypothalamic ApoA-IV elevates BAT thermogenesis and energy expenditure in lean and obese animals, and whether ApoA-IV-induced BAT thermogenesis through the activation of BAT sympathetic innervation.

## 4. Materials and Methods

### 4.1. Animals

Male ApoA-IV-KO mice and WT mice (C57BL/6J background) were generated in an AAALAC-accredited facility under conditions of controlled illumination (12:12 h light-dark cycle, lights on from 0600 to 1800 h). ApoA-IV-KO mice were back-crossed into the C57BL/6J genetic background for > 10 generations. Some WT and KO mice were bred from female and male ApoA-IV +/− heterozygote mice, and thus were littermates; other WT mice were bred from male C57BL/6J mice and female ApoA-IV +/− heterozygote mice within the same facility. All mice were genotyped by polymerase chain reaction (PCR) analysis of tail deoxyribonucleic acid (DNA) [39]. All animals at ages between 12 and 19 weeks received free access to water and either a standard chow diet (6% butter fat by weight, 59% carbohydrates by weight, and 35% protein by weight; LabDiet, St. Louis, MO, USA) or a HFD (20% butter fat by weight, 45% carbohydrates by weight, and 35% protein by weight; Research Diets, Inc., New Brunswick, NJ, USA) for 1 week. All mice were housed at 25 °C in all experiments except for cold exposure study in Experiment 4. Body weight was monitored with a top-loading balance (±0.01 g, Adenturer SL, Ohaus Corp, Pine Brook, NJ, USA). All animal protocols were approved by the Institutional Animal Care and Use Committee at Ohio University and the University of Cincinnati (15H-023 and 12/09/2021; 16H-014 and 9/15/2019).

### 4.2. Experiment 1. Energy Expenditure, RQ, and Food Intake of Chow-fed Mice

WT and ApoA-IV-KO mice (*n* = 8 per group) at age of 19 weeks had free access to a powdered chow and water for 1 week. Before the start of data collection for food intake and energy expenditure, mice were acclimatized to individual metabolic cages of an Oxymax system (Columbus Instruments, Columbus, OH, USA) for 2 days. Food intake and energy expenditure were recorded at 15-min intervals for 2 days using the manufacturer’s software. After they were transferred back to their home cages, mice continued to receive a chow diet for 3 days. On the 8th day, food was removed for 5 h before plasma, interscapular BAT, EWAT, and IWAT were collected and stored at −80 °C.

### 4.3. Experiment 2. Expression of BAT Thermogenic Protein of Mice after Acute Feeding of Dietary Lipids

WT and ApoA-IV-KO mice at 12–14 weeks of age were maintained on a chow diet. Acute or daily infusion of lipids for one week or less increases the levels of ApoA-IV [32]. To investigate whether endogenous ApoA-IV would elevate lipid-induced thermogenesis, thermogenic proteins of BAT in WT and ApoA-IV-KO mice were measured 2 h after they received an acute infusion of lipids. Briefly, after a 5-h fast, the BAT of WT and ApoA-IV-KO mice without receiving lipid feeding (*n* = 6 per group; for determination of basal level of thermogenic proteins) or 2 h after they received a 100 µL of Intralipid (20% emulsion, Sigma-Aldrich^®^, St. Louis, MO, USA) by intragastric gavage (*n* = 5 per group; for determination of lipid-induced thermogenic proteins) was collected.

### 4.4. Experiment 3. Energy Expenditure, RQ and Food Intake of Mice Fed a HFD for One Week

Because a chronic consumption of HFD for longer than 2 weeks attenuates lipid-induced production of intestinal ApoA-IV [32,33], energy expenditure and BAT thermogenesis were determined in WT and ApoA-IV-KO mice (*n* = 8 per group) at age of 19 weeks and fed a powdered HFD for 1 week. These animals were acclimatized to individual metabolic cages of an Oxymax system for 3 days, and food intake and energy expenditure were recorded at 15-min intervals for 2 days. After they were transferred back to their home cages, they continued to receive HFD for 2 days. On the 8th day, plasma, interscapular BAT, EWAT, and IWAT in 5-h fasted animals were collected for the prevention of different caloric intake involved in the regulation of BAT thermogenesis. The plasma and tissues were stored at −80 °C for further determination.

### 4.5. Experiment 4. Energy Expenditure and BAT Temperature of Chow-fed Mice during Cold Exposure

Two cohorts of animals fed with a chow diet were used to determine whole-body energy expenditure (the first cohort) and BAT temperature (the second cohort) when they were exposed to cold temperature after a 5-h fast. Mice were food deprived with only water access during the cold exposure to eliminate the cold-induced increases in food intake [5] that would affect energy expenditure and BAT temperature due to diet-induced thermogenesis [59].

The first cohort of WT (*n* = 8 per group; body weight = 23.48 ± 0.66 g) and ApoA-IV-KO mice (*n* = 8 per group; body weight = 25.36 ± 0.6 g) at 14 ± 2 weeks of age were monitored for whole-body energy expenditure in individual metabolic cages with temperature at 6.6 ± 0.5 °C for 2 h. Their energy expenditure was monitored every 20 min for last two 2 h during the cold exposure using a Comprehensive Laboratory Animal Monitoring System (Columbus Instruments, Columbus, OH, USA). The second cohort of WT (*n* = 6 per group; body weight = 24.56 ± 0.3 g) and ApoA-IV-KO mice (*n* = 6 per group; body weight = 23.96 ± 1.1 g) at 12 weeks of age received implantation of a temperature probe beneath the interscapular BAT according to our published protocol [60]. Seven days after a temperature probe implantation, WT and ApoA-IV-KO mice were fasted for 5 h. The apparent rate of decrease in norepinephrine content of BAT was the greatest in the first 3 h in response to cold exposure [42]; thus their BAT temperatures were monitored every 10 min during the 4-h cold exposure at 4 °C using a temperature transponder.

### 4.6. Thermogenic Protein and Plasma Apolipoprotein Determination

BAT protein was extracted with RIPA lysis buffer system (Santa Cruz Biotechnology, Santa Cruz, TX, USA). Plasma (4 µL) and extracted BAT protein mixed with 4x SDS sample buffer were boiled for 10 min. For the determination of UCP1 and tyrosine hydroxylase at the basal level, extracted BAT proteins (100 µg and 50 µg per lane in Figure 3A and Figure 5A, respectively) were loaded onto a 4–20% Tris-HCl gradient gel (Bio-Rad Laboratories, Hercules, CA, USA). For the determination of UCP1 and tyrosine hydroxylase induced by dietary lipids, extracted BAT proteins (10 µg per lane for UCP1 in Figure 3B and 20 µg per lane for tyrosine hydroxylase in Figure 3C) were loaded onto a 4–20% SDS gel. Gels were run at a constant voltage (100 V) until the protein standards were well separated. Proteins were then transferred to a polyvinylidene difluoride membrane (Bio-Rad Laboratories) for 2 h at a constant current of 350 mA. After blocking non-specific binding sites on the membranes for 1 h with a 5% blotting-grade blocker (Bio-Rad Laboratories) in Tris-buffer saline (TBS) with 0.1% Tween (TBS-T) solution, membranes were then incubated with one of either rabbit or mouse polyclonal antibodies in 5% bovine serum albumin (BSA): beta-tubulin (1:1000 dilution, Invitrogen^®^, IL), tyrosine hydroxylase (1:1000 dilution, Cell Signaling Technology, Danvers, MA, USA), UCP1 (1:1000 dilution, Abcam^®^, Cambridge, MA, USA), polyclonal ApoA-IV and ApoA-I antibodies (1:5000 dilution). After incubation with the primary antibody, the immunoblots were washed and then incubated with either horseradish peroxidase-conjugated goat anti-rabbit antibody or rabbit anti-mouse antibody (1:5000 dilution, Dako Cytomation, Santa Clara, CA, USA) for 1 h. Detection was achieved using the enhanced chemiluminescence system (Immobilon western chemiluminescent HRP substrate, EMD Millipore Corporation, Billerica, MA, USA). A c-Digit blot scanner (Li-Cor Biosciences, Lincoln, NE, USA) was used for development and visualization of the proteins, and infrared imaging was used for protein quantification. For BAT immunoblots, tyrosine hydroxylase and UCP1 protein concentrations were normalized to beta-tubulin. For the determination of plasma apolipoproteins, plasma ApoA-IV was normalized to ApoA-I because dietary lipid intake does not alter Apo A-I production [14].

### 4.7. Quantitative RT-PCR

When mice were maintained on a 1-week HFD, the BAT of 5-h fasted mice was collected on dry ice. Total RNA of BAT was isolated using a PureLink RNA Minikit (Invitrogen, Carlsbad, CA, USA) according to the manufacture’s protocol, and first-strand complementary DNA (cDNA) was synthesized from 1 µg total RNA using a SuperScript IV First-strand cDNA synthesis reaction kit (Invitrogen) [61]. Quantitative RT-PCR (qPCR) was performed in a 25-µL final reaction volume, including 4 µL of 10 time-diluted sample cDNA with an RT-PCR instrument (Applied Biosystems, Waltham, MA, USA) using SYBR green RT-PCR master mixes (Life Technologies, Warrington, UK). RT-PCR conditions were conducted as follows: 95 °C for 3 min for one cycle, followed by 40 cycles of 95 °C for 30 s and 60 °C for 30 s. Threshold cycle readings for each of the unknown samples were used, and the results were analyzed in Excel using the ∆∆*C*t method [34]. 36B4 mRNA levels from each sample were similar among all groups and were used as internal controls to normalize the mRNA levels. The sequences of the primers (Integrated DNA Technologies, Coralville, IA, USA) were as follows: mouse *Ucp1*, 5′-ACTGGAGGTGTGGCAGTGTTC-3′ (forward) and 5′-ACGACCTCTGTAGGCTGCCCAA-3′ (reverse); mouse *Ucp2*, 5′-TGTTGATGTGGTCAAG ACGAG AT-3′ (forward) and 5′-CATGGTAAGGGCACAGTGA-3′ (reverse); mouse *Ucp3*, 5′-AGCG GACCACTCCAGCGTC-3′ (forward) and 5′-TGAGACTCCAGCAACTTCTC-3′ (reverse); mouse *Cpt1*, 5′-ACCACTGGCCGAATGTCAAG-3′ (forward) and 5′-AGCGAGTAGCGCATGGTCAT-3′ (reverse); mouse *Ampka1*, 5′-CAGTAGGTACAC ACAGCGTAACACA-3′ (forward) and 5′-ACCTGTTACAGCAAATTCAAATGG-3′(reverse); mouse *Ampka2*, 5′-TCCAGCACAGCTGAGAA CCA-3′ (forward) and 5′-GGGATGCCGAGGACAAAGT3′(reverse); and mouse *36b4*, 5′-ATCCC TGACGCACCGCCGTG-3′ (forward) and 5′-GCGCATCATGGTGTTCTTGC -3′ (reverse).

### 4.8. Plasma Parameters

Plasma insulin and leptin levels were determined using commercial ELISA kits (Millipore, St. Charles, MO, USA). Briefly, 10 µL plasma samples were added to each well of a microtiter plate pre-coated with anti-peptide monoclonal antibodies, and the detection antibody was added. After incubation, absorbance was measured with a microplate reader (Synergy HT, BioTek Instruments, Inc., Richmond, VA, USA), and the final concentrations were calculated using standards provided with the ELISA kits. Triacylglycerol, cholesterol and glucose in the plasma were determined using Infinity triglyceride, cholesterol and glucose kits (Thermo Scientific, Middletown, VA, USA).

### 4.9. Histology of Adipocytes

WT and ApoA-IV-KO mice (*n* = 4 per group) received HFD for 1 week. BAT and IWAT of 5-h fasted animals were collected, fixed in neutral buffered 10% formalin solution overnight, embedded in paraffin, and sectioned across its extent into 7 µm in thick sections. Histology of BAT and IWAT were determined by hematoxylin and eosin (H&E) staining. Digital imagines were obtained with the 10× objective lens. Briefly, five non-overlapping parenchymal fields of each of three slides from different levels across the extent of an adipose tissue were taken light microscopy images, which were analyzed for average cell size and number using ImageJ (NIH, Bethesda, MD, USA) [60,62,63]. Briefly, five random non-overlapping microscopic fields of each section were measured. The areas of all analyzed fields were similar, around 500,000 units. The cell numbers of each field counted by ImageJ were then normalized to the same area of 500,000 units. The normalized cell numbers were presented as number of cells in unit area. The average cell size was calculated using normalized area of 500,000 units divided by normalized number of cells.

### 4.10. Statistical Analysis

All values are presented as mean ± SEM. Appropriate parametric statistical analyses and analyses of variance (ANOVA) were performed using GraphPad™ Prism (version 6.0, GraphPad Inc., San Diego, CA, USA), followed by a Sidak multiple comparisons test. Differences were considered significant if the *p* value was <0.05.

## 5. Conclusions

ApoA-IV-KO mice exhibited reduced diet-induced BAT thermogenesis and decreased energy expenditure in the dark phase. In addition, ApoA-IV-KO mice appeared normal cold-exposed BAT temperature and energy expenditure.

## Figures and Tables

**Table 1:** Body weight, adipose tissue weights, and levels of plasma parameters of WT and ApoA-IV-KO mice fed a chow diet. Tissues and plasma were collected after a 5-h fast. *n* = 7 per group. Values are represented as mean ± SEM.

Parameter	WT Mice	ApoA-IV-KO Mice
Body weight (g)	26.17 ± 0.7	27.17 ± 0.4
BAT (g)	0.17 ± 0.0	0.13 ± 0.0
EWAT (g)	0.42 ± 0.0	0.39 ± 0.0
IWAT (g)	0.27 ± 0.0	0.22 ± 0.0
Triglycerides (mg/dL)	51.86 ± 2.4	45.30 ± 4.6
Cholesterol (mg/dL)	63.78 ± 7.6	58.13 ± 2.7
Glucose (mg/dL)	108.74 ± 9.6	129.44 ± 9.1
Insulin (ng/mL)	1.08 ± 0.2	0.88 ± 0.2
Leptin (ng/mL)	1.22 ± 0.2	0.98 ± 0.3

**Table 2:** Body weight, adipose tissue weights, and levels of plasma parameters of WT and ApoA-IV-KO mice fed a high-fat diet for 1 week. Tissues and plasma were collected after a 5-h fast. *n* = 8 per group. Values are represented as mean ± SEM. * Represent significant difference relative to the WT mice (*p* < 0.05).

Sample	WT Mice	ApoA-IV-KO Mice
Body weight (g)	32.84 ± 0.74	36.30 ± 1.94
BAT (g)	0.12 ± 0.0	0.14 ± 0.0
EWAT (g)	0.57 ± 0.0	0.49 ± 0.3
IWAT (g)	0.37 ± 0.0	0.33 ± 0.0
Triglycerides (mg/dL)	48.54 ± 7.1	36.42 ± 2.0
Cholesterol (mg/dL)	148.17 ± 21.6	58.13 ± 2.7 *
Glucose (mg/dL)	133.50 ± 26.2	113.86 ± 10.8
Insulin (ng/mL)	2.61 ± 0.4	1.09 ± 0.1 *
Leptin (ng/mL)	8.73 ± 2.9	5.27 ± 0.9

**Table 3:** Number and size of adipocytes in animals fed a high-fat diet for 1 week. WT and ApoA-IV-KO mice (*n* = 4 per group) received 1 week of high-fat diet, and brown adipose tissue (BAT) and inguinal white adipose tissue (IWAT) in 5-h fasted mice were collected in 10% formalin solution. The cell number and size of BAT and IWAT of WT and ApoA-IV-KO mice were analyzed using H&E stained sections and determined using ImageJ. Cell number is number of cells within each area unit. Cell size is calculated using unit area divided by number of cells. Values represent mean ± SEM.

Cell Number and Size	WT Mice	ApoA-IV-KO Mice
Cell Number (number/unit area)		
BAT	425 ± 80.6	381 ± 29.4
IWAT	126 ± 10.7	116 ± 9.1
Cell Size (unit)		
BAT	1318 ± 240.1	1373 ± 108.6
IWAT	4298 ± 363.5	4618 ± 357.0

## Abbreviations

ApoA-IV	apolipoprotein A-IV
BAT	brown adipose tissue
HFD	high fat diet
RQ	respiratory quotient
UCP1	uncoupling protein 1
WAT	white adipose tissue
